# ADHD in the DSM-5-TR: What has changed and what has not

**DOI:** 10.3389/fpsyt.2022.1064141

**Published:** 2023-01-10

**Authors:** Athanasios Koutsoklenis, Juho Honkasilta

**Affiliations:** ^1^Department of Primary Education, Democritus University of Thrace, Alexandroupolis, Greece; ^2^Faculty of Educational Sciences, University of Helsinki, Helsinki, Finland

**Keywords:** ADHD, DSM-5-TR, revisions, American Psychiatric Association, diagnosis, diagnostic manual

## Abstract

In this article, we critically review the changes made to the DSM-5 Text Revision published in 2022 regarding the diagnostic entity of Attention Deficit/Hyperactivity Disorder (ADHD). We structure our critique around three points. The first discusses the acknowledgment of ADHD as a neurodevelopmental disorder. The second examines the definition of ADHD provided in the updated edition of the manual. The third scrutinizes the changes in the diagnostic criteria for ADHD and assesses whether these changes make the diagnosis more accurate. We conclude that DSM's latest edition does not escape the logical and scientific pitfalls of its predecessor. DSM-5-TR keeps the faith in the neo-Kraepelinian paradigm by explicitly and implicitly cultivating the essentialist medical scientific metaphor of disorder, creating the illusion that it represents scientific progress that validates ADHD as a neurodevelopmental disorder.

## 1. Introduction

From the publication of the third edition in 1980 and on, the Diagnostic and Statistical Manual of Mental Disorders (DSM) has embraced psychiatry as a branch of medicine by committing to a “neo-Kraepelinian” cause-effect biomedical framework with the assumption that biological discoveries will eventually establish the somatic etiology of separate and independent mental diseases (1). This paradigm shift was not based on promising scientific discoveries but on pragmatic consensus [see, for a discussion (2, 3)]. By the time of publishing DSM-5 in 2013, the continuous medicalization of natural human responses led by APA became increasingly critiqued within psychiatry [e.g., (3)], mental health sector [e.g., (4)], practitioners, and academia in general. Debates surrounding the critical reception of DSM-5 primarily relate to the pseudo-scientific nature of the manual and its normalizing power (5).

In this opinion paper, we critically review the changes made to the DSM-5 Text Revision published in 2022 regarding the diagnostic entity of Attention Deficit/Hyperactivity Disorder (ADHD). We structure our critique around three points. The first discusses the acknowledgment of ADHD as a neurodevelopmental disorder. The second examines the definition of ADHD provided in the updated edition of the manual. The third scrutinizes the changes in the diagnostic criteria for ADHD and assesses whether these changes make the diagnosis more accurate. We point out how DSM-5-TR keeps the faith in the neo-Kraepelinian paradigm by explicitly and implicitly cultivating the essentialist medical scientific metaphor of disorder.

## 2. Placement within “neurodevelopmental disorders”

As in its predecessor, ADHD is placed within the manual's chapter “Neurodevelopmental Disorders”. According to the DSM-5-TR neurodevelopmental disorders “are characterized by developmental deficits or differences in brain processes that produce impairments of personal, social, academic, or occupational functioning” (p. 36). The authors of the manual assert that issues relevant to the placement of ADHD have been resolved by the available data “with the preponderance of evidence supporting placement in the “Neurodevelopmental Disorders” chapter” [(6), p. 13]. This assertion is strengthened in section “Risk and Prognostic Factors”, which is more detailed than in DSM-5. The authors state that heritability is approximately 74% and that genome-wide association studies (GWAS) “have identified a number of loci enriched in evolutionarily constrained genomic regions and loss-of-function genes as well as around brain-expressed regulatory regions.” (p. 72).

However, as the authors of DSM-5-TR themselves explicitly admit, the discoveries that could confirm ADHD as a neurodevelopmental disorder have not yet materialized. Specifically, DSM-5-TR authors state that “no biological marker is diagnostic for ADHD” and that “meta-analysis of all neuroimaging studies do not show differences between individuals with ADHD and control subjects”, thus “no form of neuroimaging can be used for diagnosis of ADHD” [(6), p. 73]. Apart from what is already stated in DSM, there is no hard evidence available in the literature which proves that ADHD is a brain disorder—something that denotes a deficit in people's brains [for a discussion, see American Psychiatric Association (7), Batstra et al. (8), Schleim (9)].

The authors of DSM also leave unmentioned that the 74% heritability estimate stems from twin-studies, which as a method cannot reliably disentangle genetic from environmental factors for psychiatric presentations [see, Joseph (10)]. GWAS on the other hand yield a heritability estimate of 22%, and their suggestive findings mentioned in the manual are yet lacking convincing replication [e.g., (11)]. This challenges research to account for the ~50% gap in the assumed familial transmission of ADHD, however, the authors of DSM have remained silent about this.

The DSM-5-TR retains the same comment on the role of social context as its predecessor. More specifically, it is stated that “signs of the disorder may be minimal or absent when the individual is receiving frequent rewards for appropriate behavior, is under close supervision, is in a novel setting, is engaged in especially interesting activities, has consistent external stimulation (e.g., *via* electronic screens), or is interacting in one-on-one situations (e.g., the clinician's office)” [(6), p. 71]. It is apparent that this statement contradicts the conceptualization of ADHD provided in the manual by undermining its existence as neurodevelopmental disorder; how can frequent rewards, close supervision, novel settings and interesting activities make a neurodevelopmental disorder disappear?

Moreover, the DSM-5-TR includes a new and very interesting statement in the “Prevalence” section: “prevalence is higher in special populations such as foster children or correctional settings” [(6), p. 72]. By merely stating the fact without further discussion about psychosocial factors related to ADHD diagnoses among population living in such settings, the DSM-5-TR implies the role the alleged neurodevelopmental disorder plays in these adverse life trajectories. The likelihood that these children and young people have experienced trauma and abuse at homes [see, for example, (12, 13)] is not mentioned. Other social factors that correlate with the manifestation of inattentive, compulsive and/or hyperkinetic behaviors are not discussed, such as poverty and socioeconomic hardship (14, 15), childhood trauma (16, 17), child maltreatment (18), death in the family (19), low family cohesion (20), parental psychiatric disorder (19), parental separation (19), parental criminality (19), household dysfunction (21), familial incarceration (14) and parental long-term unemployment (22).

Leaving these factors out of the manual does not weaken the neurodevelopmental hypothesis of behaviors and functioning associated with the diagnostic category, as brain is a plastic organ which development is affected by adverse life experiences. However, silence about the complexity of the role of psychosocial factors for the development of inattentive, compulsive and hyperkinetic behaviors throughout the manual, and particularly in connection with ADHD and foster or correctional setting, implies essentialism. This bias toward biopsychological factors is strengthened in section “Risk and Prognostic Factors”, where psychosocial factors are vaguely referred to by noting that “[f]amily interaction patterns in early childhood are unlikely to cause ADHD but may influence its course or contribute to secondary development of conduct problems” [(6), p. 73]. Thus, ADHD is assumed to expose affected individuals to being vulnerable to adversities.

Essentialist neuropathological premise are also assumed in the section “Diagnostic Features”, in which it is stated that the “essential feature of attention-deficit/hyperactivity disorder (ADHD) is a persistent pattern of inattention and/or hyperactivity-impulsivity that interferes with functioning or development” [(6), p. 70]. It is difficult to comprehend how children's” own behaviors interfere with development without explicating what kinds of development trajectories are in question: neurodevelopment, school success, employment, health or what? The non-biological developmental trajectories are listed as functional consequences, thus, implying that it is neurological development that is interfered here. Understandably the logic here is that inattentive and/or hyperactive-impulsive behaviors affect ability to function, which in turn affects development, including that of the brain. However, these behaviors let alone their potential interferences with functioning imply psychosocial, societal, and sociocultural aspects of development that in turn can have biopsychological effects—not the other way around. And again, this implied assertion of neuropathology—or whatever is assumed to cause inattention and/or hyperactivity-impulsivity that interferes development—is overtly invalidated by the authors themselves by stating the lack of evidence supporting brain disorder hypotheses.

## 3. Definition

The definition of ADHD remains the same in DSM-5-TR in comparison to DSM-5. More specifically, it is stated that “ADHD is a neurodevelopmental disorder defined by impairing levels of inattention, disorganization, and/or hyperactivity-impulsivity. Inattention and disorganization entail inability to stay on task, seeming not to listen, and losing materials necessary for tasks, at levels that are inconsistent with age or developmental level. Hyperactivity-impulsivity entails overactivity, fidgeting, inability to stay seated, intruding into other people's activities, and inability to wait— symptoms that are excessive for age or developmental level” [6, p. 37]. This definition retains the circular logic of the previous edition, that is “if A then B, and if B then A.” (23). For the case of ADHD specifically, this is translated to: “if an individual has attention deficit hyperactivity disorder it is because he is inattentive, disorganized and hyperactive-impulsive, and if an individual is inattentive, disorganized and hyperactive-impulsive it is because he has ADHD.

Without concrete and objective evidence of an identifiable brain disorder there is nothing that *explains* behaviors associated with ADHD diagnosis. ADHD as a diagnostic entity remains a descriptive classification of behaviors, not an explanation for them. When behaviors are explained by using a descriptive classification, adhering to circular reasoning is inescapable. Tautology is thus inevitably disguised as scientific explanation (24). A characteristic example of this tautology is evident in the section “Functional Consequences of Attention-Deficit/Hyperactivity Disorder”. The section states that “ADHD is associated with reduced school performance and academic attainment” [(6), p. 73] which are already entailed in the diagnostic criteria in the first place. Thus, with “nothing for ADHD to be actually tied to, all that remains are observations about behavior [sic], which then act as both an indicator of, and the defining criteria for, that initial disorder” [(23), p. 251]. In circular reasoning the argument refers to nothing outside of itself.

## 4. Diagnostic criteria

The diagnostic criteria for ADHD in DSM-5-TR remained identical with those that appear in the previous edition. In a recently published paper [i.e., (25)] we have provided a thorough critique of the accuracy of DSM-5 diagnostic criteria for ADHD. To do that we have used as a blueprint the criticism for descriptive diagnoses articulated by Kirk et al. (24). In our paper we concluded that DSM-5 diagnostic criteria for ADHD are ambiguous, redundant, and arbitrary (25). We also concluded that they are ableist in the sense that they fortify normality and that they pay inadequate attention to context and agency (25). Since no changes were made in the diagnostic criteria for ADHD in the DSM-5-TR our critique can be applied as such. Therefore, we assert that no “precision” and subsequently no “enhanced precision” can be claimed for the diagnostic criteria of ADHD in the revised edition.

At this point we would also like to refer to the categories of “Other Specified Attention-Deficit/Hyperactivity Disorder” and “Unspecified Attention-Deficit/Hyperactivity Disorder”^1^ that were also present in the previous edition. Both categories apply when “symptoms characteristic of attention deficit/ hyperactivity disorder that cause clinically significant distress or impairment in social, occupational, or other important areas of functioning predominate but do not meet the full criteria for attention-deficit/hyperactivity disorder or any of the disorders in the neurodevelopmental disorders diagnostic class” [(6), p. 77]. That is, an individual is diagnosed *regardless* of not meeting the diagnostic criteria for the disorder.

In DSM-5, differences between males and females in the frequency of ADHD (more frequent in males) and the presentation of primarily inattentive features (females more likely) were briefly discussed under section “Gender-related Diagnostic Issues”. No explicit or implicit reference to potential causes or factors leading to these differences were made. DSM-5-TR presents two changes in this section. The tittle is changed to “Sex- and Gender- Related Diagnostic Issues”, making a conceptual distinction between biological notion of sex and psychological, social, historical, and cultural aspects related to biological sex (i.e., gender). Also, one sentence is added, stating that differences in “ADHD symptom severity may be due to differing genetic and cognitive liabilities between sexes” [(6), p. 73].

Thus, in contrast to the previous edition, the authors of DSM-5-TR explicitly imply the connection between inherent features and the manifestation of so-called symptoms according to sex. This attribution is strengthened by making a distinction between sex and gender yet saying nothing about gender-related factors (there is also silence about gender in “Culture-Related Diagnostic Issues” section). Instead, diagnostic issues related to sex and gender are reduced to biopsychological aspects and assumptions related to female/male binary (i.e., sex) (26). This is an example of essentialism. ADHD is portrayed as having a fixed essence (i.e., genetic, neurodevelopmental dysfunction) attributable to differences in binary sexes. This completely disregards the socially constructed roles, behaviors, expressions, and identities related to gender pluralism let alone how sociocultural aspects (e.g., gender roles, cis normativity) are intertwined with psychosocial aspects that may manifest as behaviors deemed “symptoms” [see, for example, (27)].

Culture-related normative assumptions regarding behaviors are discussed in the section about “Culture-Related Diagnostic Issues” in similar fashion to the previous version of the manual, that is, cultural bias in diagnostic practices is acknowledged. Some apparent improvements have also taken place, suggesting the need for “culturally competent diagnostic practices […] in assessing ADHD” [(6), p. 73]. In addition to the previous version, the interconnectedness of social class, race, and ethnicity in both seeking for the diagnosis for schooling (namely “non-Latinx White” parents) and affecting informant symptom rating are mentioned. Also, DSM-5-TR has been reviewed and revised by a Work Group on Ethnoracial Equity and Inclusion, which can be seen in replacement of “Latino” with “Latinx”, and in acknowledging of social oppression and racialization and their interconnectedness with diagnosing.

In line with the previous version, ADHD is portrayed as a neurodevelopmental condition within an individual caused by natural development processes over which etiology psychosocial, societal or cultural factors have no power. Instead, these factors seem to be portrayed as hindering the adequate detection and diagnosis of the condition, as evidenced in the following statement: “Underdetection may result from mislabeling of ADHD symptoms as oppositional or disruptive in socially oppressed ethnic or racialized groups because of explicit or implicit clinician bias)” [(6), p. 73]. Bluntly put, it is of importance to apply culturally competent practices in diagnosing members of socially oppressed ethnic or racialized groups to ensure correct diagnosis.

This essentialist framework of Western psychiatry guided by the DSM has long been criticized by feminist scholars within and outside psychiatry, emphasizing the intersecting links between psychological hardships and the broad social, economic and political context [see, for example, the special issue by Marecek and Gavey (28)]. While DSM-5-TR acknowledges the intersecting axes of class and racial/ethnic categorization with diagnostic judgments, it chooses to be oblivious and silent of how various social categories (e.g., gender, class, racial/ethnic) and broader contexts intersect with how behaviors and functioning develop (i.e., biopsychosocial perspective) let alone why they are diagnosed as neurodevelopmental disorders (i.e., sociocultural, and political perspectives) regardless of continues incongruence of scientific rationale and clinical practices.

## 5. Discussion

As expected, DSM's latest edition does not escape the logical and scientific pitfalls of its predecessor (e.g., circular reasoning, lack of explanatory power, accuracy related issues of diagnostic criteria etc.). What is also pervasive in the DSM-5-TR is an attempt to further solidify ADHD as a neurodevelopmental disorder. Explicitly and implicitly, DSM-5-TR creates the illusion that it represents real scientific progress that validates ADHD as a neurodevelopmental disorder. In contrast, scientific research on etiology and pathophysiology of people diagnosed with ADHD rather questions the current operationalization of ADHD as a categorical diagnosis in line with the “neo-Kraepelinian” view of discrete boundaries between health and disorder [e.g., 11]. Given DSM's multifaceted influence in organizing institutional (e.g., insurance eligibility, disability payments, educational services, legal decisions), academic (e.g., direction of research, fund allocations, course and textbook contents), and social and psychological lives (e.g., identity recognition, stigma, empathy), [e.g., (25, 29–31)], it seems unlikely that DSM would recategorize its classifications according to science it purports to adhere to.

Finally, we would like to underline the importance of the influence of the DSM since an ADHD diagnosis can expose those diagnosed to potential harm. We will briefly illustrate two examples here through the lens of the relative age effect phenomenon. First, research clearly shows an international, cross-cultural pattern of a relative-age effect in the diagnosis of ADHD [see, for a review (32)]. Children with medicalized behaviors are “railed” into certain ways of responses to those behaviors. Findings from a recent cohort study suggest that an ADHD diagnosis in childhood may not result in any improvements in quality-of-life measures in adolescents and may even negatively impact some outcomes, including the risk of self-harm (33). Second, the relative age effect phenomenon also concerns the pharmacological treatment for ADHD [see, for a review, (34)]. Children are thus exposed to the adverse effects of ADHD drugs which span from death, cardiac problems, psychotic disorders (35) to reduced appetite, difficulty sleeping, and abdominal pain (36).

On top of that, Panther et al. (37) found that most ADHD drugs prescribed for very young children were off-label, and raised concerns to lack of safety and efficacy data. The United Nations (38) has expressed concerns about the significant global increase in consumption of stimulants such as Methylphenidate (common brand names include Ritalin, Equasym, and Concerta). The report attributes this to various causes such as an increase in the number of ADHD diagnoses, misdiagnosis of ADHD, influential commercial and/or aggressive pharmaceutical marketing practices, and public pressure, such as parents' associations lobbying for their children's right to access ADHD medication [see also (39)]. In this regard, a recent meta-analysis of pediatric psychotropic drug prevalence of ADHD in the Global North reports a lack of systematic monitoring in most of the studied 23 countries (40). DSM-5-TR is likely to contribute rather than avert these trends.

## Author contributions

All authors listed have made a substantial, direct, and intellectual contribution to the work and approved it for publication.

## Footnotes

^1^The category “Other Specified Attention-Deficit/Hyperactivity Disorder” is used in situations in which the clinician chooses to communicate the specific reason that the presentation does not meet the criteria for attention-deficit/hyperactivity disorder or any specific neurodevelopmental disorder. The category ‘Unspecified Attention-Deficit/Hyperactivity Disorder' is used ‘in situations in which the clinician chooses not to specify the reason that the criteria are not met for attention deficit/hyperactivity disorder or for a specific neurodevelopmental disorder, and includes presentations in which there is insufficient information to make a more specific diagnosis [(6), p. 77].
